# Mitochondrial Functionality in Male Fertility: From Spermatogenesis to Fertilization

**DOI:** 10.3390/antiox10010098

**Published:** 2021-01-12

**Authors:** Yoo-Jin Park, Myung-Geol Pang

**Affiliations:** Department of Animal Science & Technology and BET Research Institute, Chung-Ang University, Anseong 17546, Gyeonggi-do, Korea; bealegend@cau.ac.kr

**Keywords:** mitochondria, oxidative phosphorylation, spermatozoa, male infertility, testis, epididymis, capacitation, fertilization

## Abstract

Mitochondria are structurally and functionally distinct organelles that produce adenosine triphosphate (ATP) through oxidative phosphorylation (OXPHOS), to provide energy to spermatozoa. They can also produce reactive oxidation species (ROS). While a moderate concentration of ROS is critical for tyrosine phosphorylation in cholesterol efflux, sperm–egg interaction, and fertilization, excessive ROS generation is associated with male infertility. Moreover, mitochondria participate in diverse processes ranging from spermatogenesis to fertilization to regulate male fertility. This review aimed to summarize the roles of mitochondria in male fertility depending on the sperm developmental stage (from male reproductive tract to female reproductive tract). Moreover, mitochondria are also involved in testosterone production, regulation of proton secretion into the lumen to maintain an acidic condition in the epididymis, and sperm DNA condensation during epididymal maturation. We also established the new signaling pathway using previous proteomic data associated with male fertility, to understand the overall role of mitochondria in male fertility. The pathway revealed that male infertility is associated with a loss of mitochondrial proteins in spermatozoa, which induces low sperm motility, reduces OXPHOS activity, and results in male infertility.

## 1. Introduction

**Figure 1:**
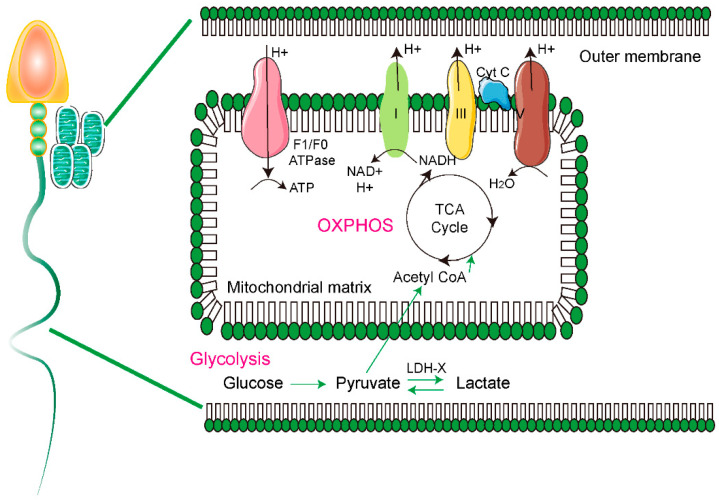
Schematic image of OXPHOS and glycolysis in spermatozoa. Pyruvate is produced by glycolysis and is then converted to lactate in the principal piece of spermatozoa by lactate dehydrogenase. Then, pyruvate in the principal piece is transported inside into the mitochondria and it is used as fuel for TCA cycles, which produce the NADH. OXPHOS take place in mitochondria of sperm midpiece. Oxidation of NADH in the electron transport chain produce the ATP molecules by OXPHOS.

Mitochondria are structurally and functionally distinct organelles that have double membranes and produce more than 90% of energy in eukaryotic cells, through oxidative phosphorylation (OXPHOS) [1,2]. The inner mitochondrial membrane, namely cristae, is a highly folded and specialized compartment that increases the membrane surface for storage of 94% of the OXPHOS complexes and adenosine triphosphate (ATP) synthase, and 85% of the total cytochrome c that is important for the OXPHOS system [3,4]. To produce ATP through OXPHOS, four respiratory enzyme complexes (complex I–IV) work in concert to create the electrochemical proton gradient in the mitochondrial inner membrane [5,6]. The tricarboxylic acid (TCA) cycle, a series of enzymatic reactions that generate acetyl coenzyme A (acetyl-CoA) by catabolism of carbohydrates, fats, and proteins, takes place in the mitochondrial matrix. Acetyl Co-A is oxidized to generate carbon dioxide (CO_2_) and reducing agents, including nicotinamide adenine dinucleotide (NADH) and flavin adenine dinucleotide (FADH_2_). NADH and FADH_2_ work as fuel for the respiratory chain, through the transfer of electrons to the mitochondrial respiratory chain for the initiation of OXPHOS (Figure 1) [7]. The flux of electrons derived from the oxidation of substrates through various redox carriers of the inner membrane electron transport chain (ETC) ultimately terminates in a four-electron reduction of molecular oxygen to water [8]. However, reactive oxidation species (ROS), such as superoxide (O_2_^−^) and hydrogen peroxide (H_2_O_2_), are produced by the incomplete reduction of oxygen by an electron [9,10]. ROS work as important secondary messengers that regulate intracellular pathways through the oxidative activation of proteins, receptors, kinases, phosphatases, caspases, ion channels, and transcription factors in normal conditions, and an imbalance between ROS and the antioxidant defense system induces oxidative stress [11,12]. Oxidative stress from ROS generation can cause various diseases, including type II diabetes, chronic inflammation, ischemia, neurodegenerative disease, and male infertility [11,13,14,15].

In spermatozoa, approximately 80 mitochondria are present in the midpiece [16]. The mitochondria’s roles are not limited to providing energy in sperm cells; they have diverse functions, including the production of steroid hormones in the testis, and regulation of cell proliferation as well as cell death for maintaining male fertility [17,18,19,20]. ROS generated from mitochondria are a double-edged sword: they are crucial for tyrosine phosphorylation in spermatozoa, cholesterol efflux, and sperm–egg interaction, but also play a role in pathological processes through oxidative stress [21]. Approximately 30–80% of male infertility cases are associated with ROS-mediated damage to spermatozoa [22]. Exposure to ROS induces damage of structural and functional components of cells such as proteins, membrane, and DNA in spermatozoa, which affects sperm motility, its ability to penetrate oocytes, and embryonic development [22,23,24]. A previous study reported that lower mitochondrial respiratory activities is closely associated with not only the reduced sperm motility, i.e., asthenozoospermia, but also lower viability and concentration of spermatozoa [25]. Moreover, lower mitochondrial membrane potential due to the abnormal morphology of the axoneme is a major cause of low sperm motility and severe asthenozoospermia [26]. Recently, comparative proteomic studies between sperm samples from infertile patients with asthenozoospermia and fertile men revealed that proteins associated with mitochondrial OXPHOS, and TCA cycles, and metabolism of pyruvate are downregulated in spermatozoa from asthenozoospermia patients [27,28]. These previous reports indicated the important role of mitochondria in sperm motility and male fertility. Another characteristic of mitochondria is the presence of their own genome (mitochondrial DNA or mtDNA) and specific ribosomes, which allow local protein synthesis [29]. Although mature spermatozoa are generally considered transcriptionally and translationally silent, Gur and Breitbart [30] provide evidence that nuclear-encoded proteins can be translated by mitochondrial-type ribosomes during sperm capacitation. Inhibition of mitochondrial translation reduced the fertilizing ability of spermatozoa by downregulating sperm motility, actin polymerization, and acrosome reactions [30]. Furthermore, mitochondria are highly plastic organelles that can modulate their localization and shape through fusion and fission [31]. The interaction between mitochondrial fusion protein 2 (MFN2) and mitostatin-related protein 1 (MNS1) regulates the biogenesis of sperm tails, which are associated with sperm motility [32]. Numerous studies have suggested that mitochondria participate in various crucial processes that regulate male fertility. Therefore, in this review, we discuss the diverse roles of mitochondria in male fertility, depending on the sperm developmental stage (spermatogenesis to fertilization).

## 2. Mitochondria Dynamics in the Testis

### 2.1. Metabolic and Morphological Changes in Mitochondria during Spermatogenesis

Spermatogenesis is a complex process that produces fully differentiated spermatozoa and occurs in highly convoluted seminiferous tubules of the testis. This process can be divided spatiotemporally into three major steps: (1) extensive mitotic division to amplify spermatogonial stem cells (SSCs); (2) meiosis to reduce the number of chromosomes to produce haploid spermatids; and (3) spermiogenesis for morphological transformation of round spermatids into spermatozoa [33]. To maintain the proliferation and differentiation of SSCs, the mammalian target of rapamycin (mTOR) is activated in testis for supporting the nutritional support of spermatogenesis [34]. During spermatogenesis, the number and morphology of mitochondria in male germ cells change, depending on the differentiation status of the cells [35]. Spermatogonia and early spermatocytes (meiotic phase I) contain small and orthodox mitochondria that have low OXPHOS activity because their mitochondrial matrix is an expanded, less dense, compact cristae compartment [36]. Otherwise, late spermatocytes, spermatids, and spermatozoa contain the more elongated and condensed form of mitochondria that is suitable for the structure of sperm flagellum [37]. Although the number of mitochondria is reduced following the loss of cytoplasm during spermiogenesis, the condensed mitochondria are helically anchored around the midpiece of the sperm tail and become more efficient metabolically [19,38].

**Figure 2:**
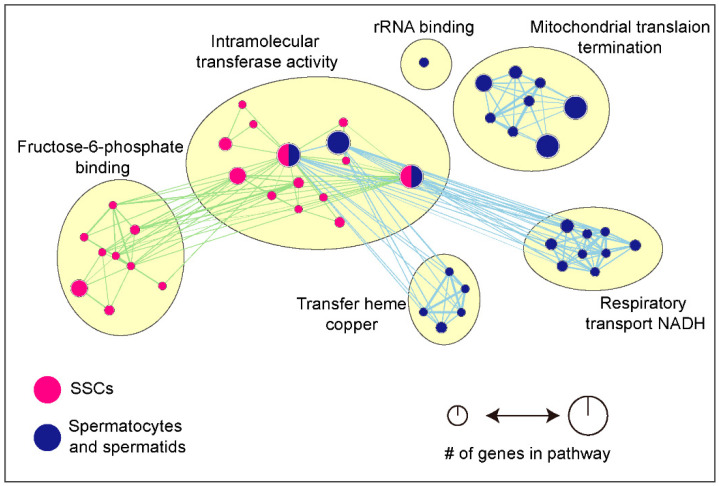
Signaling Pathways According to Differentially Expressed Genes between Spermatogonia and Spermatocytes/Spermatids. Molecular functions based on the differentially expressed genes in spermatogonia (SSCs) and differentiated male germ cells (spermatocytes and spermatids) were determining using g: Profiler, Cytoscape, and EnrichmentMap. Purple represents enrichment in spermatogonia, while blue represents enrichment in differentiated male germ cells. Node size indicated how many genes are involved to pathways.

Metabolic profiling and mitochondrial morphology change along with the differential microenvironment in the testis, and this microenvironment contains SSCs, spermatocytes, and spermatids. Especially, testis is an immune-privileged organ that maintains the immune homeostasis by the blood–testis barrier (BTB). The BTB is a specialized tight junction formed by Sertoli cells and it provides a unique microenvironment in the adluminal compartment of the seminiferous epithelium for preventing autoantigens residing in spermatogenic cells from being recognized by host immune cells [39,40]. SSCs are located in the basal compartment of the BTB, which means that SSCs can use blood-derived nutrients and glucose for ATP production [41]. Moreover, SSCs contain a small number of immature mitochondria which are highly vacuolated spherical structures with few cristae and a low electron lucid matrix. Otherwise, spermatocytes and spermatids contain a large number of mature mitochondria with a dense matrix compartment and a wide cristae [42,43]. In addition, a recent review has reported that the expression of glycolysis-related genes, such as those encoding hexokinase-1, enolases (ENOs), phosphofructokinase, pyruvate dehydrogenase kinase 2, and Myc, was higher in SSCs than in spermatocytes and spermatids [42]. Low oxygen concentration, called a hypoxic condition, enhances the glycolytic state by stimulation of the transcription factor, which is associated with glycolytic enzymes. And oxygen availability to cells decreases OXPHOS and TCA rates, and contributes to a decrease in respiration rate. Therefore, it is suggested that a low oxygen microenvironment stimulates the glycolysis, which is strongly associated with regulation of stem cell self-renewal and regenerative capacity [42,44]. Spermatocytes, which are located in the adluminal compartment of the seminiferous tubule, contain mature mitochondria and have a lower glycolytic potential than SSCs [45]. The major source for metabolism of these differentiated cells is lactate provided by Sertoli cells [46]. Previous studies have suggested that protein synthesis in round spermatids can be stimulated rather by lactate than glucose, because the production rate of ATP, which is the major factor of protein synthesis, through lactate utilization is faster than that of glucose [47]. Moreover, lactate suppress the male germ cell apoptosis through the activation of Fas receptor signaling pathways regardless of ATP presence [41,48]. Furthermore, owing to the demand for higher rates of ATP production and the higher concentration of oxygen during differentiation of SSCs, OXPHOS capability is increased in differentiated cells by the upregulation of ETC complex-related genes. These genes include those encoding NADH: ubiquinone oxidoreductase core (NDUF) subunits, which are related to an electrochemical gradient of complex IV during OXPHOS, cytochrome c oxidase (COX) subunits, and COX assembly factors (COAs) that are associated with the respiratory chain [49,50]. An increase in OXPHOS capability during spermatogenesis is directly linked to the upregulation of aerobic metabolism via a higher rate of oxygen consumption, the generation of a large number of ATP, and the downregulation of glycolytic enzymes [51,52,53]. Other important changes in mitochondria during developmental transitions are mitochondrial augmentation and maturation, associated with an increase in mitochondrial respiratory chain complex assembly and the development of cristae [42]. Following the differentiation process, there is an increase in mitochondrial proteins associated with mitochondrial organization and mtDNA replication [54,55,56,57]. In most eukaryotic cells, mitochondria are continuously remodeled by mitochondrial fusion and fission for the quality control of cells [58]. Defects in the mitochondrial fusion process induces mitochondrial divergence and reduction of mtDNA and OXPHOS [59]. Mitochondrial fusion and fission in cells play an important role in cell proliferation and differentiation through the elimination of damaged mitochondria and the introduction of maximized ATP production [58,60]. Mitochondrial fusion proteins, known as mitofusins (MFNs), are required for the remodeling of mitochondrial morphology to produce elongated mitochondria with condensed cristae and for the upregulation of OXPHOS during the transition of SSCs to spermatocytes. However, MFNs are unessential for the self-renewal of spermatogonia [61,62,63]. Finally, an increase in OXPHOS during differentiation is linked to the upregulation of mtDNA replication-related genes, including DNA polymerase subunit gamma, solute carrier family 25 member 33, ribonuclease H1, mitochondrial genome maintenance exonuclease 1 ribonucleoside-diphosphate reductase subunit M2 B, and those encoding mitochondrial DNA polymerase [55]. Moreover, mtDNA mutations induce meiotic arrest at the zygotene stage of meiotic prophase I, which enhances apoptosis, thereby resulting in male infertility [18]. Based on the differentially expressed genes during spermatogenesis reported in previous studies (Table 1) [42,54,55,56], we tried to summarize molecular function-related signaling pathways using g:Profiler, Cytoscape, and EnrichmentMap, according to the protocol of Reimand et al. [64] (Table 2 and Figure 2) To identify the powerful and significant signaling pathways, false discovery rate (FDR) was applied in this study. FDR is one of the widely used statistical methods to identify the significance in the genome-wide expression data analysis [65]. We identified six signaling pathways linked to fructose-6-phosphate binding, intramolecular transferase activity, rRNA binding, mitochondrial translation termination, respiratory transport NADH, and transfer heme copper (FDR < 0.05). Similar to previous reports, fructose-6-phosphate binding involved a signaling pathway associated with improvement of cell reprogramming efficiency and proliferation through the activation of glycolysis [66,67,68], and is highly expressed in spermatogonia (Figure 2). Moreover, the mitochondrial translation termination-related signaling pathway and respiratory chain in mitochondria-associated signaling pathways, including respiratory transport NADH and transfer heme copper, were detected as being highly expressed following sperm differentiation (Figure 2). In summary, glycolytic activity is linked to enhanced cellular proliferation and the maintenance of long-term regeneration of SSCs, and the metabolic transition from glycolysis to OXPHOS is likely to be a key driver in spermatogonial differentiation.

### 2.2. Energy Metabolism in Germ Cell Response to Testicular Somatic Cells Depending on the Sperm Developmental State

Leydig and Sertoli cells are testicular somatic cells that play critical roles in supporting spermatogenesis. Seminiferous tubule, the site of spermatogenesis, is compartmentalized into basal and adluminal parts of the lumen by tight junctions of Sertoli cells; these parts are termed “BTB” [69]. BTB supports the immunological barrier in regulating infiltration by immune cells and cytokines in the seminiferous tubules, and regulates the movement of substances between the seminiferous tubules and blood [70,71,72]. In addition, Sertoli cells work as nurse cells to establish an adequate luminal environment in the seminiferous tubules by providing energy and nutritional support for the development of male germ cells to mature sperm cells [41,73]. Sertoli cells are a major energy source for the spermatogenesis in the testis. They secrete lactate, which is produced primarily by glucose metabolism, but can also be produced by metabolism of lipids, amino acids, and glycogen [74,75,76,77]. Briefly, glucose is transported into the Sertoli cells by the glucose transporter 1, and it goes through the sequential reaction of glycolysis to produce the pyruvate, as the end-of product of glycolysis. Pyruvate is metabolized to lactate by lactate dehydrogenase, which is regulated by growth factors, cytokines, and sex steroid hormones [78,79,80]. Lactate is then exported into the germ cells via monocarboxylate transporter 1 for use as a germ cell energy source. Although why the germ cells use lactate from Sertoli cells rather than glucose as their major energy source is unknown, Boussouar and Benahmed [41] suggested that because development of germ cells into the sperm is a highly activated process which requires the sufficient energy source, germ cells rely predominantly on lactate metabolism for ATP production rather than glucose. Furthermore, adenosine monophosphate (AMP)-activated protein kinase (AMPK) is closely related to lactate production through the upregulation of glucose uptake from glucose transporters [81,82]. Lactate is converted to pyruvate, which is transported across the inner mitochondrial membrane via the mitochondrial pyruvate carrier (MPC) to support germ cell differentiation [41,83]. Because of the metabolic shift from glycolysis to OXPHOS during germ cell differentiation, MPC is more highly expressed in spermatocytes to facilitate OXPHOS using pyruvates than in spermatogonia [61]. Moreover, Sertoli cells regulate the number of germ cells via regulation of apoptosis during mitosis and prevent excessive proliferation of germ cells [84]. Sertoli cells can produce ATP through lipid β-oxidation following phagocytosis of apoptotic spermatogenic cells. Apoptotic cells undergo degradation after phagocytosis and convert to lipids, resulting in a drastic increase in long-chain acyl-CoA dehydrogenase (LACD) in the mitochondria of Sertoli cells [85]. Finally, Sertoli cells secrete activin A to modulate mitochondrial structures in the condensed form and facilitate OXPHOS during differentiation through the regulation of transcriptional events associated with mitochondrial biogenesis by modulation of early transcriptional regulators including peroxisome proliferator-activated receptor-γ coactivator 1α, nuclear respiratory factor (NRF) 1, and NRF2 [41,86]. Activation of transcriptional events in mitochondria by activin A requires the higher energy consumptions through the electron transport to serve proton gradient and the oxidative phosphorylation [87].

Although Leydig cells are the major somatic cells in the testis, there is relatively little information about its role in male germ cell differentiation, compared to Sertoli cells. Leydig cells are located in the basal compartment in the testis and produce steroid hormones, such as androgens, primarily testosterone, by circulating luteinizing hormone (LH) secreted by the pituitary [88]. Testosterone regulates spermatogenesis through the regulation of adhesion of round spermatids to Sertoli cells and release of spermatids from testis [89]. LH stimulates cyclic AMP (cAMP) production in Leydig cells, to accelerate the transport of cholesterol to the inner mitochondrial membrane. In the inner mitochondrial membrane, cholesterol is converted to pregnenolone by P450 cholesterol side-chain cleavage enzyme (encoded by *CYP11A1*), which is the first step in the production of testosterone [90]. It is noteworthy that the main role of cholesterol in mitochondria is ATP production; however, it cannot participate in ATP production in Leydig cells because the specific morphology of cristae, in close proximity to the mitochondrial membrane, does not allow the introducing the F1 complexes of ATP synthase on the matrix side of the cristae [91].

Altogether, testicular somatic cells provide structural and functional support for the spermatogenesis. Specifically, high glycolytic activity in Sertoli cells is linked to the accessibility of germ cells to oxygen depending on their location in testicular compartmentalization. It also reduces ROS-related damage by low metabolism, to maintain stem cells [19].

## 3. Orchestrating Mitochondria in the Epididymis

### 3.1. Epididymal Epithelial Cells Work in a Concerted Manner to Establish Optimal Conditions for Sperm Maturation

After leaving the testis, spermatozoa undergo maturation to acquire their fertilizing ability during epididymal transit [92,93]. Specialized epididymal epithelial cells including narrow, clear, principal, and basal cells, coordinate to maintain the unique microenvironment, acidic pH, and low bicarbonate concentration in the epididymal lumen, and prevent the pre-activation of spermatozoa during epididymal maturation [92,94,95]. The apical membrane and intracellular vesicles in clear and narrow cells are rich in proton-pumping vacuolar ATPases (V-ATPases), which secrete protons into the lumen to support the acidic pH in the epididymis [92,94]. Basal cells are the sensors of the luminal environment. In narrow and clear cells, V-ATPase is activated by nitric oxide secreted by basal cells, through stimulation of angiotensin-II type 2 receptor (AGTR2) by angiotensin [96]. In addition, principal cells reabsorb bicarbonate through the cystic fibrosis transmembrane conductance regulator (CFTR) and secrete proteins and sodium/hydrogen exchangers (NHEs) [97,98].

**Figure 3:**
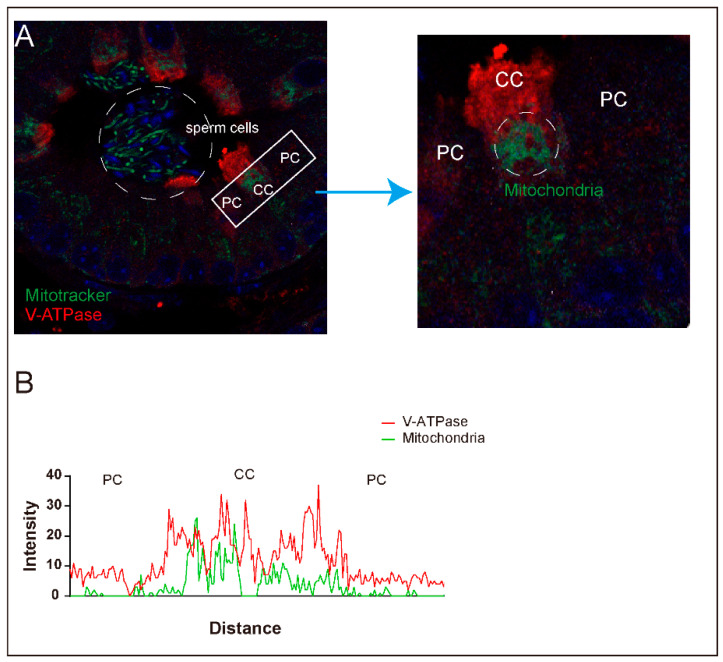
Different distribution of mitochondria between clear cells and other cells in the epididymis. Mitochondria were labelled with a Mitotracker (Green) and clear cells (CCs) were labelled with V-ATPase (Red). Representative images (**A**) show the different distribution of mitochondria between clear cells and other cells, such as principal cells (PCs, unlabeled cells). The fluorescence intensity results (**B**) indicated that mitochondria are more enriched in clear cells than principal cells. The images were obtained using Nikon Eclipse Ni-U equipped with a Nikon DS-Ri2 camera and the Nikon NIS-Elements Fr software (Version 5.11, Nikon Instruments Inc., Melville, NY, USA).

Mitochondria-rich cells, including intercalated cells in the kidney, and osteoclasts, have specific phenotypes, such as the ability to extend from the membrane as microplicae, high expression of proton-pumping ATPase, highly activated endocytosis and exocytosis of V-ATPase-containing vesicles, rod-shaped intramembranous particles in the plasma membrane and intracellular vesicles, and high levels of cytosolic carbonic anhydrase type II [99]. In addition, narrow and clear cells in the epididymis are classified as mitochondria-rich cells that contain more mitochondria than the adjacent epithelial cells, such as principal cells (Figure 3) [99]. Narrow and clear cells revealed the same aspects as other mitochondria-rich cells. The abundant mitochondria in these cells are closely associated with luminal acidification through the regulation of carbonic anhydrase activity, endocytotic activity, and proton secretion by V-ATPase in the epididymis [99,100].

### 3.2. Regulation of ROS in the Epididymis for Sperm Maturation

During sperm passage through the epididymis, the composition of the plasma membrane in spermatozoa is remodeled by acquiring or shedding proteins through the secretory and absorptive activities of epididymal epithelial cells, resulting in complete maturation of the lipids of spermatozoa [101]. In particular, the proportion of polyunsaturated fatty acids (PUFAs) increased in the plasma membrane of the sperm following epididymal maturation. Although PUFAs increase the membrane integrity and fusion ability of oocytes for fertilization, they are also closely linked with an increase in susceptibility to oxidative stress in spermatozoa [101,102]. When the production of ROS exceeds the capacity of the antioxidant defense system, PUFA-containing spermatozoa undergo lipid peroxidation [103].

Another important change in spermatozoa during testicular and epididymal maturation is chromatin condensation, which protects sperm DNA from mechanical and biological stresses before fertilization [104]. The following are the two major steps for condensing the sperm DNA: replacement of histone by protamine during spermiogenesis, and formation of disulfide bond between protamine thiol groups during epididymal maturation [104,105,106]. Thiol oxidation by ROS forms disulfide bonds, which is comparable to the activation of antioxidant enzyme activities, such as glutathione peroxidases (GPXs) and peroxiredoxins (PRDXs) [107,108,109,110]. Moreover, GPXs, including GPX3, GPX4, and GPX5, can be detected in the epididymis and play important roles in sperm maturation. GPX3 is an androgen-dependent cytosolic enzyme that is localized in the epididymis and vas deferens [111,112]. GPX5 is highly expressed in the epididymal epithelial cells, secreted into the lumen, and bound to the acrosomal region of spermatozoa. Furthermore, GPX5 is recovered during the transition of the spermatozoa in the female reproductive tract [113,114]. Finally, GPX4 is detected not only in the nucleus and mitochondria of spermatozoa, but also in epididymal epithelial cells, and is closely related to the progressive increase in sperm DNA compaction during the transition from caput to cauda epididymis [108,115,116]. In addition, GPX4 is the major component of mitochondrial protein in the sperm midpiece, and its activation is positively correlated with sperm motility, integrity of morphology, viability, and consequently, with male fertility [117].

Other major antioxidant enzymes are PRDXs, which are thiol-dependent peroxidases and are classified into three groups: PRDX6 (monomer), PRDX1–4 (dimer), and the atypical 2-Cys PRDX5 (monomer). This classification is based on the cysteine residues in the active sites of the enzymes [118,119,120]. The major role of PRDXs is associated with ROS, especially H_2_O_2_ and scavengers, and PRDXs act as regulators of ROS-dependent signaling [121,122]. In the epididymis, PRDX1 and PRDX6 showed different expression levels depending on the epididymal segments, indicating the differential role of PRDXs during sperm maturation [123]. Particularly, PRDX6 plays an important role in sperm fertility through the regulation of DNA condensation, prevention of lipid peroxidation, and repair of membrane lipid peroxidation in spermatozoa during epididymal maturation [118,124,125]. Moreover, exposure to oxidative stress upregulates the expression of PRDXs in epididymal spermatozoa during maturation, in order to reduce oxidative damage [123].

In summary, epithelial cells work in harmony to provide appropriate environmental conditions for sperm maturation using the following mechanisms: (1) regulation of protein secretion/absorption from epithelial cells for remodeling of sperm plasma membrane; (2) upregulation of antioxidant enzyme activities for sperm DNA condensation; and (3) ROS modulation to reduce oxidative stress in spermatozoa during epididymal transit.

## 4. Mitochondria Is a Key Organelle for Sperm Fertility

### 4.1. Mitochondrial Activity of Spermatozoa in Female Reproductive Tract

To fertilize an oocyte, spermatozoa must undergo a capacitation process that controls dynamic changes in sperm morphology such as remodeling of plasma membrane to facilitate the penetration to oocytes and function during their transition in the female reproductive tract [126]. Capacitation, as a post-translational modification, is associated with tyrosine phosphorylation, which is the activation of tyrosine kinase by cAMP. This activation increases membrane fluidity, calcium influx, and hyper-activated motility, and these changes facilitate the penetration of spermatozoa into the oocytes [127,128,129,130]. ATP is required for the successful capacitation and acquisition of hyper-activated motility, and these are regulated by two major metabolic pathways, including glycolysis and mitochondrial OXPHOS [131,132,133,134]. Glycolysis is compartmentalized in the principal piece of spermatozoa and is the main source of ATP for supporting motility. OXPHOS is a more efficient pathway than glycolysis for the production of ATP, and occurs in the mitochondrial-rich midpiece of spermatozoa. OXPHOS is essential not only for supporting hyper-activated motility, but also for regulating the acrosome-reaction and chromatin integrity in spermatozoa [19,135]. Mitochondrial morphology is transformed during capacitation towards being more loosely wrapped around the axoneme following an increase in the mitochondrial volume, which is associated with flagellar beat frequency [136,137]. These results indicate that morphological changes in the mitochondria are associated with hyperactive sperm motility during capacitation. Finally, the most important role of OXPHOS in spermatozoa is ROS generation. One of the important cellular modification of spermatozoa during capacitation is protein tyrosine phosphorylation, which is regulated by ROS [138]. ROS stimulates the adenylyl cyclase (AC) for producing of cyclic adenosine monophosphate (cAMP) from ATP. Subsequently, cAMP activates protein kinase A (PKA) that triggers not only ROS production by stimulation of NADPH oxidase in a fibrous sheath, but also phosphorylation of serine and tyrosine residues for activation of protein tyrosine kinase (PTK). PTK activates the phosphorylation of tyrosine residues of fibrous sheath in the axoneme of sperm flagellum, which can introduce hyperactivated motility of spermatozoa [138,139]. Moreover, following the ROS stimulation, intracellular calcium ions generated during capacitation are released from the acrosome to induce the cleavage of phosphatidylinosital-4,5-biphosphate (PIP2), which forms diacylglycerol (DAG) and inositol triphosphate (IP3). DAG stimulates protein kinase C (PKC), which lead to an influx of calcium ions and phosphorylation of phospholipase A2 (PLA2). Phosphorylated PLA2 increase the membrane fluidity of spermatozoa, which plays an important role in the regulation of the acrosome reaction to facilitate the sperm–oocyte fusion [140,141,142]. Therefore, a moderate amount of ROS in spermatozoa is essential for sperm capacitation, acrosome reaction, and fertilization; however, excessive ROS production in spermatozoa affects male fertility by increasing lipid peroxidation and DNA damage [143,144]. Moreover, excessive ROS reduce the intracellular ATP concentration, which causes a diminishing of the flagella beat frequency and phosphorylation in spermatozoa, and results in the loss of sperm motility [143,145,146]. Therefore, the maintenance of an appropriate ROS level through OXPHOS in spermatozoa is pivotal for sperm capacitation and fertilization.

### 4.2. Mitochondrial Proteins Associated with Male Fertility

Up to 15% of couples are infertile in worldwide, and half of these cases are derived from male infertility factors, including defects in sperm morphology and motility [147]. Approximately 60% of idiopathic male infertility is closely related to decreased sperm motility [148,149]. Mitochondria are the major regulators of sperm motility; however, they are also fundamental for capacitation, acrosome reaction, and fertility through the phosphorylation of mitochondrial proteins including signaling proteins associated with chaperones, structure, and metabolism of spermatozoa [150]. Especially, phosphorylation of sperm-surface chaperones stimulates the structural modifications to facilitate the sperm-zona interaction [151]. Therefore, knowledge of the roles of mitochondria in male fertility remains one of the most important issues in understanding the mechanisms of male fertility. Recently, comparative “omic” studies (proteomics and transcriptomics) have been conducted between fertile, sub-fertile, and infertile spermatozoa in varied mammalian species, including bovine, porcine, and human species [27,152,153,154,155,156,157,158,159,160,161]. Among the varied omic studies of spermatozoa related to male fertility, we consolidated the diverse proteomic studies in spermatozoa from infertile patients according to their pathological conditions such as asthenozoospermia, idiopathic, varicocele, and ROS damage [156,157,160,161,162,163,164,165], to identify the comprehensive fertility-related signaling pathways. Between the two groups, 315 proteins showed different expression patterns; 181 proteins were highly expressed in spermatozoa from fertile patients, while 134 proteins were abundant in spermatozoa from infertile patients (Table 3). To analyze the cellular processes regulated by differentially expressed proteins, pathway enrichment analysis was conducted using g: Profiler, Cytoscape, and EnrichmentMap according to Reimand’s report [64].

**Figure 4:**
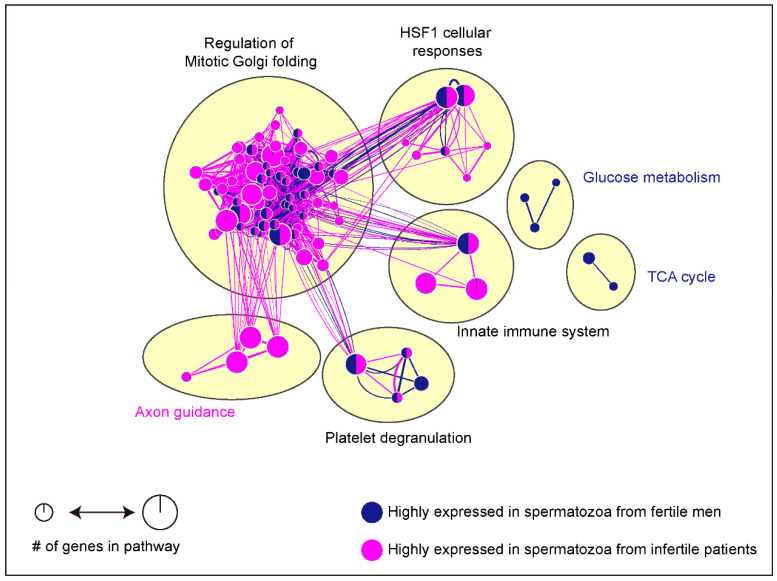
Signaling pathways according to differentially expressed genes between fertile and infertile human spermatozoa. Signaling pathways based on the differentially expressed proteins in spermatozoa from fertile and infertile men were determined using g: Profiler, Cytoscape, and EnrichmentMap. Blue represents enrichment in spermatozoa from fertile men, while purple represents enrichment in spermatozoa from infertile patients. Node size indicated how many genes are involved with pathways.

Seven signaling pathways related to the regulation of mitotic Golgi folding, heat shock factor 1 cellular response, innate immune system, platelet degranulation, axon guidance, glucose metabolism, and TCA cycle were identified (FDR < 0.01, Figure 4). Only one signaling pathway, axon guidance, was closely linked with highly expressed proteins including TUBA1C, TUBA3C, TUBB4B, LAMC1, MMP9, GPC1, MYH9, MYL6, A0A087WVQ6, HSPA8, RPLP2, RPS27A, TUBA3E, ACTB, HSP90AA1, TUBB8, HSP90AB1, TUBB3, TUBA4A, MYL12A, TUBA1B, PSMB3, KRT10, KRT1, KRT9, and ACTB in spermatozoa from infertile patients (Table 4). Axon guidance is well known as the navigation of growing axons to reach their target cells in a contact-dependent manner during neural development [166]. Moreover, axon guidance is closely related to apoptosis through the activation of caspases by the signals from the mitochondria following the response to external pro-apoptotic signals or the removal of trophic support molecules [167,168]. Furthermore, axon guidance is closely linked with enhancement of chemoattraction by sAC-dependent cAMP and PKA activation to regulate the recruitment of deleted in colorectal cancer [169,170]. Although it is very difficult to find studies investigating the role of axon guidance in male infertility, the previous studies support the two possible mechanisms. First, overexpression of the axon guidance signaling pathway-related proteins stimulates the apoptosis by activation of caspase cascade in spermatozoa during fertilization, resulting in male infertility. Secondly, overexpression of the axon guidance signaling pathway-related proteins may interrupt the chemotaxis of sperm towards eggs through the dysregulation of chemoattractant of spermatozoa during fertilization. Otherwise, two signaling pathways associated with energy production, such as glucose metabolism and TCA cycle, were closely linked with highly expressed proteins in spermatozoa from fertile men (Figure 4). Glucose metabolism was a significant pathway associated with ENO1, PGK2, PRKACA, NUP35, TPI1, GAPDHS, and ALDOA and TCA cycle was closely related to LDHC, ACO2, NDUFS1, OGDH, UQCRC2, IDH3B, MPC2, and ACAD9 (Table 4, FDR < 0.05). Glycolysis is the previous step to supply the pyruvate to mitochondria for the initiation of TCA cycle, and these two steps play an important role in providing energy for sperm motility and viability [171,172]. As mentioned above, we established the fertility-related signaling pathways based on a protein list from fertile and infertile patients from asthenozoospermic patients, ROS damaged associated infertility, idiopathic, IVF failed, and varicocele. Glucose metabolism and TCA cycle signaling pathway-related proteins were highly expressed in spermatozoa from fertile men, or rather, the proteins were defected in spermatozoa from infertile patients. Therefore, we postulated that defects of energy metabolism-related proteins in spermatozoa may be a major cause of male infertility regardless of infertility type.

### 4.3. Elimination of Paternal Mitochondrial DNA during Fertilization

According to studies, mtDNA is predominantly inherited from the mother in most animal taxa because paternal mitochondria are removed during fertilization [173,174]. Although rare, a coexistence of paternal and maternal mtDNA is possible and is associated with mitochondrial disease in humans [175]. Shitara et al. [176] found that transmission of the paternal mtDNA is restricted only in the F1 generation from an interspecific cross, while the transmission of paternal mtDNA did not occur in F1 from backcrosses. These results indicated that species-specific regulation of paternal mtDNA rejection in fertilized oocytes ensures the rigorously maternal inheritance of mtDNA.

There are several hypothetical mechanisms for the removal of paternal sperm-borne mitochondria during fertilization. First is the dilution effect derived from the relatively low number of mitochondria in spermatozoa when compared to that in oocytes. Second, the dilution of paternal alleles could be amplified by a mitochondrial bottleneck, which is a process that selectively eliminates the defective mtDNA in oocytes [175,177]. The third hypothesis is that there is no mtDNA or degraded mtDNA present in fertilizing spermatozoa because the mitochondria in spermatozoa are degraded before reaching the oocytes [178,179]. It has been proposed in the fourth hypothesis that degradation of sperm-borne mitochondria occurs in oocytes following sperm mitophagy associated with the ubiquitin-proteasome system [180,181]. After spermatozoa enter the oocyte, sperm-contributed mitochondria and mtDNA were tagged with ubiquitin for degradation by the ubiquitin-proteasome system [182]. Moreover, ubiquitination of prohibitin, the major protein of the inner mitochondrial membrane, occurs during spermatogenesis and is closely associated with the recognition of sperm mitochondria by the ubiquitin-proteasome-dependent proteolytic machinery in oocytes after fertilization [183]. Taken together, these reports provide a better understanding of sperm-borne mtDNA degradation associated with male fertility and mitochondrial diseases.

## 5. Conclusions

Overall, this review highlights the comprehensive roles of mitochondria from spermatogenesis to fertilization. The major function of mitochondria is ATP production through OXPHOS during the process of male reproduction, that is, from spermatogenesis to fertilization. However, mitochondria can be used to modulate numerous processes, including (1) SSC differentiation, testicular somatic cell development, and testosterone production in the testis; (2) luminal acidification and sperm DNA condensation in the epididymis; and (3) ROS homeostasis for sperm capacitation and acrosome reaction in the female reproductive tract. Moreover, this review suggests a fertility-related comprehensive signaling pathway and showed that the defects of energy metabolism-related proteins may be the cause of varied infertility associated with motility defects, ROS damage, varicocele, and unknown factors.

## Figures and Tables

**Table 1:** Summary of differentially expressed genes between spermatogonia and spermatocytes/spermatids.

Highly Expressed Genes in Spermatogonia	Highly Expressed Genes in Spermatocytes and Spermatids
22-oxoglutarate dehydrogenase-like, mitochondrial (OGDHL); 6-phosphofructo-2-kinase/fructose-2,6-bisphosphatase 3 (PFKFB3); Aminoacyl tRNA synthase complex-interacting multifunctional protein 1 (AIMP1), ATP-dependent 6-phosphofructokinase, liver type (PFKL); ATP-dependent 6-phosphofructokinase, muscle type (PFKM); Enolase (ENO) 3; ENO4, Fructose-bisphosphate aldolase A (ALDOA); GDH/6PGL endoplasmic bifunctional protein (H6PD); Glyceraldehyde-3-phosphate dehydrogenase (GAPDH); Glycosylphosphatidylinositol (GPI); Myc proto-oncogene protein (MYC); Nischarin (NISCH); Phosphoglucomutase-2 (PGM2); Phosphoglycerate mutase 1 (PGAM1); Pyruvate dehydrogenase (acetyl-transferring) kinase isozyme 2, mitochondrial (PDK2); Pyruvate dehydrogenase E1 component subunit beta, mitochondrial (PDHB); Triosephosphate isomerase (TPI1)	39S ribosomal protein L53, mitochondrial (MRPL53); Complex I assembly factor ACAD9, mitochondrial (ACAD9); Cytosolic carboxypeptidase 1 (AGTPBP1); Apoptosis-inducing factor 1, mitochondrial (AIFM1); Polyamine-transporting ATPase 13A2 (ATP13A2); ATP synthase subunit epsilon, mitochondrial (ATP5E); ATP5G2; ATP5J; ATP5L; Aurora kinase A-interacting protein (AURKAIP1); Mitochondrial chaperone BCS1 (BCS1L); Coiled-coil-helix-coiled-coil-helix domain-containing protein 5 (CHCHD5); Clustered mitochondria protein homolog (CLUH); Cytochrome c oxidase assembly factor 1 homolog (COA1); COA5; Cytochrome c oxidase copper chaperone COX19 (COX19); COX20; COX5A; COX6C; COX7B; COX17; Cytochrome c oxidase subunit NDUFA4 (NDUFA4); 28S ribosomal protein S29, mitochondrial (DAP3); DnaJ homolog subfamily A member 3, mitochondrial (DNAJA3); Mitochondrial fission 1 protein (FIS1); Frataxin, mitochondrial (FXN); Elongation factor G, mitochondrial (GFM1); GFM2; Golgi phosphoprotein 3 (GOLPH3); Histidine—tRNA ligase, cytoplasmic 1 (HARS1); HARS2; Probable leucine—tRNA ligase, mitochondrial (LARS2); 39S ribosomal protein L1, mitochondrial (MRPL1); MRPL14; MRPL18; MRPL19; MRPL20; MRPL24; MRPL3; MRPL30; MRPL44; MRPL47; MRPL55; 28S ribosomal protein S18b, mitochondrial (MRPS10); MRPS14; MRPS15; MRPS17; MRPS18A; MRPS18C; MRPS21; MRPS33; MRPS5; Transcription termination factor 4, mitochondrial (MTERF4); Mitochondrial ubiquitin ligase activator of NFKB 1 (MUL1); NADH dehydrogenase [ubiquinone] 1 alpha subcomplex subunit 1 (NDUFA1); NDUFA11; NDUFA12; NDUFA13; NDUFA2; NDUFA3; NDUFA4; NDUFA5; NDUFA6; NDUFA7; NDUFAF2; NDUFAF3; NDUFAF7; NDUFB1; NDUFB2; NDUFB3; NDUFB4; NDUFB7; NDUFB8; NDUFC1; NDUFC2; NDUFS1; NDUFS2; NDUFS5; NDUFS6; NDUFS7; Nitric oxide-associated protein 1 (NOA1); Presenilins-associated rhomboid-like protein, mitochondrial (PARL); Glutamyl-tRNA(Gln) amidotransferase subunit A, mitochondrial (QRSL1); Small integral membrane protein 20 (SMIM20); Sequestosome-1 (SQSTM1); Dimethyladenosine transferase 2, mitochondrial (TFB2M); Ubiquinol-cytochrome-c reductase complex assembly factor 2 (UQCC2); Cytochrome b-c1 complex subunit 9 (UQCR10)

**Table 2:** Summary signaling pathways based on differentially expressed genes in male germ cells during spermatogenesis.

	Molecular Functions	FDR	Related Genes
Highly expressed genes in Spermatogonia	Glycolysis	<0.001	GPI, TPI1, PGAM1, PFKL, PFKFB3, ENO3, PFKM, GAPDH
	Glucose metabolism	<0.001	GPI, TPI1, PGAM1, PFKL, PFKFB3, ENO3, PFKM, GAPDH
	Metabolism of carbohydrates	<0.001	GPI, TPI1, PGAM1, PFKL, PFKFB3, ENO3, PFKM, GAPDH, PGM2
	Gluconeogenesis	<0.001	GPI, TPI1, PGAM1, ENO3, GAPDH
	Metabolism	<0.001	GPI, TPI1, PGAM1, PFKL, PFKFB3, ENO3, PFKM, GAPDH, PGM2, PDK2, AIMP1, PDHB
	Regulation of pyruvate dehydrogenase (PDH) complex	<0.05	PDK2, PDHB
Highly expressed genes in Spermatocytes and Spermatids	Respiratory electron transport	<0.001	NDUFA4, NDUFS5, NDUFB7, NDUFA6, NDUFC2, NDUFA13, NDUFC1, NDUFA12, NDUFB1, NDUFB2, NDUFB3, NDUFA5, NDUFB4, NDUFA2, NDUFAF7, NDUFA1, NDUFAF3, NDUFS6, ACAD9, NDUFS2, NDUFS1, NDUFS7, NDUFA3, NDUFA7, NDUFAF2, NDUFA11, UQCR10, COX20, COX7B, COX5A, COX6C
	Respiratory electron transport, ATP synthesis by chemiosmotic coupling, and heat production by uncoupling proteins	<0.001	NDUFA4, NDUFS5, NDUFB7, NDUFA6, NDUFC2, NDUFA13, NDUFC1, NDUFA12, NDUFB1, NDUFB2, NDUFB3, NDUFA5, NDUFB4, NDUFA2, NDUFAF7, NDUFA1, NDUFAF3, NDUFS6, ACAD9, NDUFS2, NDUFS1, NDUFS7, NDUFA3, NDUFA7, NDUFAF2, NDUFA11, UQCR10, COX20, COX7B, COX5A, COX6C, ATP5J
	Complex I biogenesis	<0.001	NDUFS5, NDUFB7, NDUFA6, NDUFC2, NDUFA13, NDUFC1, NDUFA12, NDUFB1, NDUFB2, NDUFB3, NDUFA5, NDUFB4, NDUFA2, NDUFAF7, NDUFA1, NDUFAF3, NDUFS6, ACAD9, NDUFS2, NDUFS1, NDUFS7, NDUFA3, NDUFA7, NDUFAF2, NDUFA11
	The citric acid (TCA) cycle and respiratory electron transport	<0.001	NDUFA4, NDUFS5, NDUFB7, NDUFA6, NDUFC2, NDUFA13, NDUFC1, NDUFA12, NDUFB1, NDUFB2, NDUFB3, NDUFA5, NDUFB4, NDUFA2, NDUFAF7, NDUFA1, NDUFAF3, NDUFS6, ACAD9, NDUFS2, NDUFS1, NDUFS7, NDUFA3, NDUFA7, NDUFAF2, NDUFA11, UQCR10, COX20, COX7B, COX5A, COX6C, ATP5J
	Mitochondrial translation	<0.001	MRPL53, AURKAIP1, GFM2, DAP3, MRPS17, MRPS18C, MRPS15, MRPS18A, MRPS21, MRPL47, MRPL1, MRPL3, MRPS33, MRPS14, MRPS10, MRPS5, MRPL30, MRPL20, MRPL24, MRPL14, GFM1, MRPL19, MRPL18, MRPL55, MRPL44
	Mitochondrial translation termination	<0.001	MRPL53, AURKAIP1, GFM2, DAP3, MRPS17, MRPS18C, MRPS15, MRPS18A, MRPS21, MRPL47, MRPL1, MRPL3, MRPS33, MRPS14, MRPS10, MRPS5, MRPL30, MRPL20, MRPL24, MRPL14, MRPL19, MRPL18, MRPL55, MRPL44
	Mitochondrial translation elongation	<0.001	MRPL53, AURKAIP1, DAP3, MRPS17, MRPS18C, MRPS15, MRPS18A, MRPS21, MRPL47, MRPL1, MRPL3, MRPS33, MRPS14, MRPS10, MRPS5, MRPL30, MRPL20, MRPL24, MRPL14, GFM1, MRPL19, MRPL18, MRPL55, MRPL44
	Mitochondrial translation initiation	<0.001	MRPL53, AURKAIP1, DAP3, MRPS17, MRPS18C, MRPS15, MRPS18A, MRPS21, MRPL47, MRPL1, MRPL3, MRPS33, MRPS14, MRPS10, MRPS5, MRPL30, MRPL20, MRPL24, MRPL14, MRPL19, MRPL18, MRPL55, MRPL44
	Translation	<0.001	MRPL53, AURKAIP1, GFM2, DAP3, MRPS17, MRPS18C, MRPS15, MRPS18A, HARS2, MRPS21, MRPL47, LARS2, MRPL1, MRPL3, MRPS33, MRPS14, MRPS10, MRPS5, MRPL30, MRPL20, MRPL24, MRPL14, GFM1, MRPL19, MRPL18, MRPL55, MRPL44
	Metabolism	<0.001	NDUFA4, NDUFS5, NDUFB7, NDUFA6, NDUFC2, NDUFA13, NDUFC1, NDUFA12, NDUFB1, NDUFB2, NDUFB3, NDUFA5, NDUFB4, NDUFA2, NDUFAF7, NDUFA1, NDUFAF3, NDUFS6, ACAD9, NDUFS2, NDUFS1, NDUFS7, NDUFA3, NDUFA7, NDUFAF2, NDUFA11, UQCR10, COX20, COX7B, COX5A, COX6C, ATP5J
	Metabolism of proteins	<0.001	MRPL53, AURKAIP1, GFM2, DAP3, MUL1, AGTPBP1, MRPS17, MRPS18C, MRPS15, MRPS18A, HARS2, MRPS21, MRPL47, LARS2, MRPL1, MRPL3, MRPS33, MRPS14, MRPS10, MRPS5, MRPL30, MRPL20, MRPL24, MRPL14, GFM1, MRPL19, MRPL18, MRPL55, MRPL44

**Table 3:** Differentially expressed proteins in spermatozoa from fertile and infertile men according to their pathological conditions.

Highly Expressed Proteins in Spermatozoa from Fertile Men	Highly Expressed Proteins in Spermatozoa from Fertile Men	Pathological Condition
CSE1 chromosome segregation 1-like protein (CSE1L); Beta actin (ACTB); Chaperonin containing TCP1, subunit 8 (theta); Clathrin heavy chain 1 (CLTC); Enolase 1 (ENO1); Eukaryotic translation elongation factor 2 (EEF2); Fibronectin 1 isoform 3 preproprotein (FN1); Heat shock 70 kda protein 2 (HSPA2); Heat shock 90 kda protein 1, alpha isoform 1 (HSP90AA1); Heat shock protein beta-1 (HSPB1); L-lactate dehydrogenase C (LDHC); Peptidylprolyl isomerase A (PPIA); Peroxiredoxin 6 (PRDX6); Phosphoglycerate kinase 2 (PGK2); Prolactin-induced protein (PIP); Prostate specific antigen isoform 1 preproprotein (KLK3); Pyruvate kinase, muscle isoform M2 (PKM); Semenogelin I isoform a preproprotein (SEMG1); Semenogelin II precursor (SEMG2); Sorbitol dehydrogenase (SORD); Tubulin, beta 4 (TUBB4A); Tyrosine 3/tryptophan 5 -monooxygenase activation protein, zeta polypeptide (YWHAZ); Valosin-containing protein (VCP)	Acetyl-Coenzyme A acetyltransferase 1 precursor (ACAT1); Acid phosphatase, prostate short isoform precursor (ACPP); Angiotensin I converting enzyme 1 isoform 1 precursor (ACE); ATP synthase, H + transporting, mitochondrial F1 complex, alpha subunit precursor (ATP5A1); Brain creatine kinase (CKB); Chaperonin containing TCP1, subunit 4 (CCT4); Chaperonin containing TCP1, subunit 5 (CCT8); Chromosome 20 open reading frame 3 (c20orf3); Clusterin preproprotein (CLU); Eukaryotic translation elongation factor 1 alpha 1 (EEF1A1); Fru fumarate hydratase precursor (FH); Glutamine synthetase (GLUL); Glutathione peroxidase 4 isoform A precursor (GPX4); Glutathione S-transferase mu 3 (GSTM3); Glyceraldehyde-3-phosphate dehydrogenase (GAPDH); Heat shock 70 kda protein 5 (HSPA5); Heat shock protein 90 kda beta, member 1 (HSP90B1); Histone cluster 1, h2aa (HIST1H2AA); Histone cluster 1, h2ba (HIST1H2BA); Lactotransferrin precursor (LTF); Mitochondrial ATP synthase beta subunit precursor (ATP5B); Mitochondrial malate dehydrogenase precursor (MDH2); Olfactomedin 4 precursor (OLFM4); Outer dense fiber of sperm tails 2 isoform 2 (ODF2); Phosphoglycerate dehydrogenase (PHGDH); Phospholipase A2, group IIA precursor (PLA2G2A); Protein disulfide-isomerase A3 precursor (PDIA3); RAB2A, member RAS oncogene family (RAB2A); Ropporin (ROPN1); Ropporin, rhophilin associated protein 1B (ROPN1B); Saccharopine dehydrogenase (SCCPDH); Sperm autoantigenic protein 17 (SPA17); Sperm protein associated with the nucleus, X chromosome, family member C (SPANX); Transglutaminase 4 (TGM4); Triosephosphate isomerase 1 isoform 1 (TPI1); Tubulin alpha 6 (TUBA1C); Tubulin, alpha 3c (TUBA3C); Tubulin, beta, 2 (TUBB4B); Voltage-dependent anion channel 2 (VDAC2)	Oxidative stress [164]
AIG2-like domain 1(A2LD1); EF-hand calcium binding domain (RCN2); F-box protein 2 (FBXO2); Cell division cycle 34 homolog, ubiquitin-conjugating enzyme E2R2 (CDC34, UBE2R2); Calumenin (CALU); Regulator of chromosome condensation 1(RCC1); ATPase, Na+/K+ transporting, beta 3 polypeptide (ATP1B3); Progesterone receptor membrane component 1/2 (PGRMC1/ PGRMC2); Polyribonucleotide nucleotidyltransferase 1(PNPT1); Acyl-coa thioesterase 7(ACOT7); Acrosomal vesicle protein 1(ACRV1); Fragile X mental retardation 1 neighbor (FMR1NB); Cysteine-rich secretory protein 2/3 (CRISP2/CRISP3)	Actin, alpha 1, skeletal muscle (ACTA1); Abhydrolase domain containing 2 (ABHD2); Phosphofructokinase, muscle (PFKM); Beta-2-microglobulin (B2M); Protein phosphatase 5(PPP5C)	IVF failed [165]
Clusterin (CLU); RNA-binding regulatory subunit of oncogene DJ1 (PARK7); Prostate-specific antigen (KLK3); Prolactin induced protein (PIP); Superoxide dismutase 1 (SOD1); Semenogelin-2 (SEMG2); ATP synthase subunit δ, mitochondrial (ATP5D); 78-kda glucose-regulated protein precursor (HSPA5); Aspartate-rich protein 1 (DRICH1); Nucleoporin p58/p45 (NUP58); Protein c9orf135 (c9orf135); Coiled-coil domain-containing protein 42 (CCDC42); 5’-deoxynucleotidase HDDC2 (HDDC2); Protein DPCD (DPCD); Heterogeneous nuclear ribonucleoprotein M (HNRNPM); Syntaxin-12 (STX12); Acrosin-binding protein (ACRBP); Adenylate kinase 7 (AK7); Sodium/potassium-transporting atpase subunit alpha-4 (ATP1A4); T-complex protein 1 subunit zeta-2 (CCT6B); Heat shock 70kda protein 2, isoform CRA_a (HSPA2); Heat shock 70 kda protein 4L (HSPA4L); Nucleoside diphosphate kinase (NME5); Ruvb-like helicase (EC 3.6.4.12); (RUVBL1); Sperm autoantigenic protein 17 (SPA17); Camp-dependent protein kinase catalytic subunit alpha (PRKACA); Truncated camp-dependent protein kinase A type 1A regulatory subunit (PRKAR1A); Aconitate hydratase, mitochondrial (ACO2); NADH-ubiquinone oxidoreductase 75 kda subunit, mitochondrial (NDUFS1); 2-oxoglutarate dehydrogenase, mitochondrial (OGDH); Cytochrome b-c1 complex subunit 2, mitochondrial (UQCRC2); Apolipoprotein A-I, isoform CRA_a (APOA1); V-type proton atpase subunit E 1 (ATP6V1E1); Isocitrate dehydrogenase [NAD] subunit, mitochondrial (IDH3B I); 2-oxoglutarate dehydrogenase, mitochondrial (ODO1); Short-chain acyl-coa dehydrogenase (ACADS); Long-chain-fatty-acid--coa ligase 6 (ACSL6); Delta(3,5); -Delta(2,4); -dienoyl-coa isomerase, mitochondrial (ECH1); Voltage-dependent calcium channel subunit alpha-2/delta-2 (CACNA2D2); Glucosidase, alpha acid (Pompe disease, glycogen storage disease type II); isoform CRA_a (GAA); cAMP-dependent protein kinase type I-alpha regulatory subunit (PRKAR1A)	Gelsolin (GSN); Prostatic acid phosphatase precursor (ACPP); Integrin alpha-M (ITAM); Integrin beta-2 (FINC); Outer dense fiber protein 2 (ODFP2); Protein-glutamine gamma-glutamyltransferase 4 (TGM4); Filamin-B (FLNB); Tektin-3 (TEKT3); Cytosolic non-specific dipeptidase (CNDP2); Ras gtpase-activating-like protein IQGAP1 (p195); (IQGA1); Azurocidin (CAP7); ATP- citrate synthase (ACLY); Glutamine--fructose-6-phosphate aminotransferase (GFPT1)	Varicocele [162,163]
Cullin-associated NEDD8-dissociated protein 1 (CAND1); Endoplasmin precursor (ENPL); Sperm equatorial segment protein 1 precursor (SPESP); Semenogelin-2 precursor (SEMG2); Semenogelin-1 preproprotein (SEMG1); 3’(2’); 5’-bisphosphate nucleotidase 1 isoform X3 (BPNT1); Mitochondrial import receptor subunit TOM40 homolog (TOM40); Protein FAM71B (FA71B); L-amino-acid oxidase isoform 2 precursor (OXLA); Izumo sperm-egg fusion protein 3 precursor (S4R3E6); Sperm surface protein Sp17 (SP17); Diablo homolog, mitochondrial isoform 1 precursor (DBLOH); Solute carrier family 2, facilitated glucose transporter member 14 isoform a (GTR14); Mitochondrial pyruvate carrier 2 isoform X1 (MPC2); Multifunctional protein ADE2 isoform 2 (PUR6); Thioredoxin domain-containing protein 3 (TXND3); Plasma serine protease inhibitor preproprotein (IPSP); Mannose-6-phosphate isomerase isoform 1 (MPI); NADH dehydrogenase [ubiquinone] 1 alpha subcomplex subunit 11 isoform 1 (NDUAB); UV excision repair protein RAD23 homolog B isoform 1 (RD23B); Cysteine-rich secretory protein 1 isoform 1 precursor (CRIS1); Four and a half LIM domains protein 1 isoform 5 (FHL1); ADP-ribosylation factor 1 (ARF1); Fibronectin isoform 1 preproprotein (Q6MZM7); 3-mercaptopyruvate sulfurtransferase isoform 1 (THTM); Leucine zipper transcription factor-like protein 1 isoform 1 (LZTL1); Glucosamine-6-phosphate isomerase 2 isoform 1 (GNPI2); Calcium-binding mitochondrial carrier protein Aralar2 isoform 1 (CMC2); Dynactin subunit 2 isoform 2 (DCTN2); Mimitin, mitochondrial (NDUF2); 26S protease regulatory subunit 7 isoform 1 (PRS7); Ubiquitin carboxyl-terminal hydrolase isozyme L1 (UCHL1); Protein phosphatase 1A isoform 3 (PPM1A); 26S protease regulatory subunit 6A (PRS6A); Inactive serine protease 54 precursor (PRS54); Valine--trna ligase (SYVC); EGF-like repeat and discoidin I-like domain-containing protein 3 isoform 1 precursor (EDIL3); Acyl-coa dehydrogenase family member 9, mitochondrial (ACAD9); Matrix-remodeling-associated protein 5 precursor (MXRA5); Lon protease homolog, mitochondrial isoform 1 precursor (LONM); Redox-regulatory protein FAM213A isoform 1 precursor (PXL2A); Translocation protein SEC63 homolog (SEC63); Alpha-soluble NSF attachment protein (SNAA); Exportin-2 isoform 1 (XPO2); Dehydrogenase/reductase SDR family member 7B (DRS7B); Calcium-binding mitochondrial carrier protein Aralar1 (CMC1); SUN domain-containing protein 3 isoform 2 (SUN3); 26S protease regulatory subunit 8 isoform 1 (PRS8); Tetratricopeptide repeat protein 25 (TTC25); Casein kinase I isoform alpha isoform 3 (KC1A); Dynactin subunit 1 isoform 5 (DCTN1); NADPH--cytochrome P450 reductase (NCPR); Syntaxin-12 (STX12); Nucleoporin NUP53 isoform a (NUP35); Extracellular matrix protein 1 isoform 1 precursor (ECM1); Ribonuclease 4 precursor (RNAS4); Calcium/calmodulin-dependent protein kinase type IV (KCC4); Fibronectin isoform 3 preproprotein (Q6MZF4); Maestro heat-like repeat-containing protein family member 7 (MROH7); Short/branched chain specific acyl-coa dehydrogenase, mitochondrial precursor (ACDSB); Choline transporter-like protein 5 isoform B (CTL5); 26S protease regulatory subunit 6B isoform 1 (PRS6B); Protein canopy homolog 3 precursor (CNPY3); Carboxypeptidase B preproprotein (CBPB1); CMT1A duplicated region transcript 15 protein (CDRTF); Dolichyldiphosphatase 1 isoform a (DOPP1); Serotransferrin precursor (TRFE); Acetyl-coa acetyltransferase, cytosolic (THIC); Mitochondrial coenzyme A transporter SLC25A42 isoform X1 (S2542); Arrestin domain-containing protein 5 (ARRD5); Aquaporin-7 (AQP7)	Protein NDRG1 (NDRG1); Laminin subunit gamma-1 precursor (LAMC1); Succinyl-coa:3-ketoacid coenzyme A transferase 2, mitochondrial precursor (SCOT2); Cytosolic non-specific dipeptidase isoform X2 (CNDP2); Testis-specific serine kinase substrate (TSKS); Matrix metalloproteinase-9 preproprotein (MMP9); Myoferlin isoform b (MYOF); Alpha-actinin-1 isoform c (ACTN1); Prostate stem cell antigen preproprotein (D3DWI6); Thioredoxin-related transmembrane protein 1 precursor (TMX1); Glypican-1 precursor (GPC1); Septin-4 isoform X1 (B4E0R1); CD177 antigen precursor (CD177); Cytoplasmic dynein 1 heavy chain 1 (DYHC1); Olfactomedin-4 precursor (OLFM4); Sorbitol dehydrogenase (DHSO); Laminin subunit beta-2 precursor (LAMB2); Myosin-9 (MYH9); Annexin A4 (ANXA4); Histone H1.3 (H13); Platelet-activating factor acetylhydrolase isoform X1 (PAFA); Glycerol kinase 2 (GLPK2); Annexin A1 (ANXA1); Integrin alpha-M isoform 1 precursor (ITAM); Peroxiredoxin-2 (PRDX2); Neprilysin isoform X1 (NEP); Dynein heavy chain 7, axonemal (DYH7); Glutamate carboxypeptidase 2 isoform 1 (FOLH1); Myeloperoxidase precursor (PERM); Annexin A5 (ANXA5); Ectonucleotide pyrophosphatase/phosphodiesterase family member 3 (ENPP3); Nucleoporin p58/p45 isoform a (NUP58); Annexin A2 isoform 2 (ANXA2); Dynein heavy chain 17, axonemal (DYH17); Voltage-dependent anion-selective channel protein 1 isoform X3 (VDAC1); Mitochondrial inner membrane protein isoform 2 (MIC60); Annexin A3 (ANXA3); Myosin light polypeptide 6 isoform 1 (MYL6); Lysosome-associated membrane glycoprotein 2 isoform C precursor (LAMP2); A-kinase anchor protein 3 (AKAP3); Aminopeptidase N isoform X1 (AMPN); Galectin-3-binding protein precursor (LG3BP); Carcinoembryonic antigen-related cell adhesion molecule 1 isoform 1 precursor (CEAM1); Nardilysin isoform a (NRDC); Peroxiredoxin-1 (PRDX1); Protein sel-1 homolog 1 isoform 1 precursor (SE1L1); Clathrin heavy chain 1 isoform 2 (A0A087WVQ6); Nuclear pore membrane glycoprotein 210 precursor (PO210); L-xylulose reductase isoform 2 (DCXR); A-kinase anchor protein 4 isoform 2 (AKAP4); Pyruvate kinase PKM isoform b (KPYM); Prostate-specific antigen isoform 1 preproprotein (KLK3); Mitochondrial carrier homolog 2 (MTCH2); Stress-70 protein, mitochondrial precursor (GRP75); Tubulin beta-4B chain (TBB4B); Prostatic acid phosphatase isoform TM-PAP precursor (PPAP); Lactotransferrin isoform 1 precursor (TRFL); Tubulin alpha-3C/D chain (TBA3C); Actin, cytoplasmic 2 (ACTG); Nuclear pore membrane glycoprotein 210-like isoform 1 precursor (P210L); Presequence protease, mitochondrial isoform 2 precursor (PREP); T-complex protein 1 subunit zeta isoform a (TCPZ); Protein disulfide-isomerase A4 precursor (PDIA4); T-complex protein 1 subunit theta isoform 1 (TCPQ); Short-chain specific acyl-coa dehydrogenase, mitochondrial precursor (ACADS); Dihydrolipoyllysine-residue acetyltransferase component of pyruvate dehydrogenase complex, mitochondrial precursor (ODP2); Protein disulfide-isomerase precursor (PDIA1); Very long-chain specific acyl-coa dehydrogenase, mitochondrial isoform 3 (ACADV)	Idiopathic [161]
Actin gamma enteric smooth muscle (ACTG2); Actin alpha skeletal muscle (ACTA1); Sperm protein associated with the nucleus on the X chromosome B F (SPANXB1); Cathelicidin antimicrobial peptide (CAMP); POTE ankyrin domain family member E (POTEE); Phospholipid hydroperoxide glutathione peroxidase mitochondrial (GPX4); Outer dense fiber protein 1 (ODF1); Clusterin (CLU); 78 kDa glucose regulated protein (HSPA5); Heat shock related 70 kDa protein 2 (HSPA2); Prolactin inducible protein (PIP); Glutathione S transferase Mu 3 (GSTM3); Tubulin beta 6 chain (TUBB6); Tubulin alpha 8 chain (TUBA8); Lactotransferrin (LTF); Triose phosphate isomerase (TPI1); A kinase anchor protein 4 (AKAP4); Glyceraldehyde 3 phosphate dehydrogenase, testis specific (GAPDHS); ATP synthase subunit beta mitochondrial (ATP5B); A kinase anchor protein 3 (AKAP3); Ras related protein Rab 2A (RAB2A); Tubulin alpha 3C D chain (TUBA3C); Ropporin 1B (ROPN1B); Histone H2B type 1 A (HIST1H2BA); Fructose bisphosphatealdolase A (ALDOA); Elongation factor 1 alpha 2 (EEF1A2); Acrosin binding protein (ACRBP); Elongation factor 1 gamma (EEF1G); Histone H2A type 1 A (HIST1H2AA); Dynein light chain 1 cytoplasmic (DYNLL1); Epididymal sperm binding protein 1 (ELSPBP1); Acrosomal protein SP 10 (ACRV1); Actin alpha cardiac muscle 1 (ACTC1)	glyceraldehyde-3-phosphate dehydrogenase, testis-specific (G3PT); Keratin type I cytoskeletal 10 (KRT10); Heat shock 70 kDa protein 1 like (HSPA1L); Tubulin beta 2C chain (TUBB2C); Heat shock cognate 71 kDa protein (HSPA8); 60S acidic ribosomal protein P2 (RPLP2); Creatine kinase B type (CKB); Prostate specific antigen (KLK3); Ubiquitin (RPS27A); Elongation factor 1 alpha 1 (EEF1A1); Tubulin beta 4 chain (TUBB4); Tubulin alpha 3E chain (TUBA3E); Keratin type II cytoskeletal 1 (KRT1); Hsc70 interacting protein (ST13); Heat shock 70 kDa protein 6 (HSPA6); Keratin type I cytoskeletal 9 (KRT9); Prostatic acid phosphatase (ACPP); Sorbitol dehydrogenase (SORD); Heat shock 70 kDa protein 1A 1B (HSPA1A); Ras related protein Rab 2B (RAB2B); Putative heat shock protein HSP 90 beta 3 (HSP90AB3P); Putative heat shock protein HSP 90 beta 4 (HSP90AB4P); Endoplasmin (HSP90B1); Beta 2 microglobulin (B2M); Putative heat shock protein HSP 90 alpha A2 (HSP90AA2); POTE ankyrin domain family member F (POTEF); Putative heat shock protein HSP 90 beta 2 (HSP90AB2P); 14 3 3 protein zeta delta (YWHAZ); Actin cytoplasmic 1 (ACTB); Heat shock protein beta 1 (HSPB1); Heat shock protein HSP 90 alpha (HSP90AA1); Beta actin like protein 2 (ACTBL2); Tubulin beta 8 chain (TUBB8); Heat shock protein HSP 90 beta (HSP90AB1); Tubulin beta 3 chain (TUBB3); Neutrophil defensin 1 (DEFA1); Putative elongation factor 1 alpha like 3 (EEF1AL3); Tubulin beta chain (TUBB); Tubulin alpha 4A chain (TUBA4A); Phospholipase A2 membrane associated (PLA2G2A); Myosin regulatory light chain 12A (MYL12A); Rho GDP dissociation inhibitor 1 (ARHGDIA); Tubulin alpha 1B chain (TUBA1B); Clusterin precursor (CLUpre);Dihydrolipoamide dehydrogenase (DLD); precursor (DLDpre); Fumarate hydratase precursor (FHpre); Heat shock-related 70 kDa protein 2 (HSPA2); Inositol-1(or 4); -monophosphatase (IMPA1); 3-mercapto-pyruvate sulfurtransferase/Delta 3,5-delta 2,4-dienoyl-CoA isomerase precursor (MPST/ ECH1pre); Proteosome beta 3 subunit human (PSMB3); Semenogelin I protein precursor (SEMG1pre); Testis-expressed sequence 12 protein (TEX12)	Astheozoopsermic [156,157]

**Table 4:** Summary of signaling pathways associated with fertility- and infertility-related proteins in human spermatozoa.

	Molecular Functions	FDR	Related Genes
Fertility	Glucose metabolism	<0.001	ENO1, PGK2, PRKACA, NUP35, TPI1, GAPDHS, ALDOA
	TCA cycle	<0.05	LDHC, ACO2, NDUFS1, OGDH, UQCRC2, IDH3B, MPC2, ACAD9
Infertility	Axon guidance	<0.001	TUBA1C, TUBA3C, TUBB4B, LAMC1, MMP9, GPC1, MYH9, MYL6, A0A087WVQ6, HSPA8, RPLP2, RPS27A, TUBA3E, ACTB, HSP90AA1, TUBB8, HSP90AB1, TUBB3, TUBA4A, MYL12A, TUBA1B, PSMB3, KRT10, KRT1, KRT9, ACTB
